# Supplementary material for: Protective effects of stem cells from human exfoliated deciduous teeth derived conditioned medium on osteoarthritic chondrocytes
Source: PLoS One. 2020 Sep 4;15(9):e0238449. doi: 10.1371/journal.pone.0238449 (PMC7473555; doi:10.1371/journal.pone.0238449)
Supplement: S1 File — (DOCX) [file pone.0238449.s001.docx]

**Flow cytometry histogram of MSC phenotype**

CCM

SFM (48 h)

SFM (72 h)

Isotype control

Isotype control

CCM

Isotype control

Isotype control

SFM (48 h)


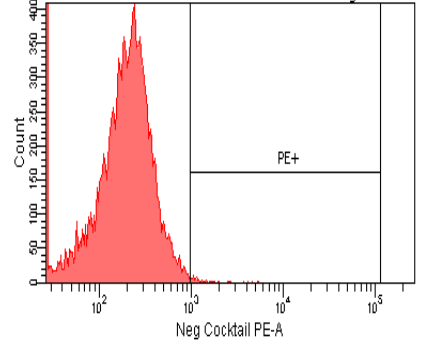


SFM (72 h)
