# Supplementary material for: Protective effects of stem cells from human exfoliated deciduous teeth derived conditioned medium on osteoarthritic chondrocytes
Source: PLoS One. 2020 Sep 4;15(9):e0238449. doi: 10.1371/journal.pone.0238449 (PMC7473555; doi:10.1371/journal.pone.0238449)
Supplement: Attachment — Submitted filename: Response to reviewers.docx [file pone.0238449.s002.docx]

**Academic Editor**

**Comment**

Thank you for submitting your manuscript to PLOS ONE. After careful consideration, we feel that it has merit but does not fully meet PLOS ONE’s publication criteria as it currently stands. Therefore, we invite you to submit a revised version of the manuscript that addresses the points raised during the review process.

This paper is of interest pending some minor amendments that have been specified by each of the two referees. Authors must follow all their concerns and amend figures and text in red in their revised text.

**Response**

Thank you for finding merit in our manuscript. The concerns raised by the referees are addressed in the revised version as recommended.

**Reviewer #1:**

In this paper Authors evaluated the regenerative effect of stem cells from human exfoliated deciduous teeth (SHED) conditioned medium (CM) on OA chondrocytes.
The paper is interesting and well organized and the experiments are well performed. Some minor changes are needed.

**Response**

Thank you for the commendation and observations made which aimed to improve the paper.

**Comment**
English language should be revised.

**Response**

English language has been thoroughly checked and improved as suggested.

**Comment**
In figure 1 images should be presented to a better resolution.

**Response**

Figure 1 resolution was improved as recommended.

**Comment**
Authors should adjust histograms: they should have the same layout (bold and normal characters, border…)

**Response**

Histograms have been adjusted as suggested in the revised version

Reviewer #2:

This study was designed to assess how conditioned medium (CM)
from SHED could modulate tissue microenvironment of catabolic processes in OA chondrocytes. The manuscript is interesting, but there are some points that need to be addressed.

**Response**

Thank you for the observations aimed at improving our manuscript.

**Comment**
MMP13 must be evaluated by western blotting.

**Response**

Western blotting of MMP13 was not reported in the current study. However, MMP-13 level was assessed using ELISA after treatment of OA chondrocytes with CM and was earlier presented in Fig 5a.

**Comment**

Table 2 must be deleted or added as supplemental file and the data must be reported as histogram.

**Response**

The reason we excluded the histogram for Table 2 was to avoid the results being presented twice since they were derived from the histogram. There are three groups of different surface proteins, which would make the results awkward for the readers if presented in histograms. Based on this, we have retained the results presented in Table 2 and provided the histogram in the supplementary file 1.

**Comment**
In figure 2, OCT4 is distributed in cytoplasm. Usually, in stem cell, it is nuclear. Clarify this point.

**Response**

The explanation on the localization of OCT4 in the cytoplasm is provided in the revised version with supporting literature. OCT4 is a nucleocytoplasmic shuttling protein as previously reported.

**Comment**
The English language must be revised by native English speaker.

**Response**

The English language has been extensively revised as suggested in the amended version.
